# Next Generation Sequencing and Electromyography Reveal the Involvement of the *P2RX6* Gene in Myopathy

**DOI:** 10.3390/cimb46020073

**Published:** 2024-01-29

**Authors:** Mirella Vinci, Girolamo Aurelio Vitello, Donatella Greco, Simone Treccarichi, Alda Ragalmuto, Antonino Musumeci, Antonio Fallea, Concetta Federico, Francesco Calì, Salvatore Saccone, Maurizio Elia

**Affiliations:** 1 Oasi Research Institute—IRCCS, 94018 Troina, Italy; mvinci@oasi.en.it (M.V.); avitello@oasi.en.it (G.A.V.); dgreco@oasi.en.it (D.G.); streccarichi@oasi.en.it (S.T.); aragalmuto@oasi.en.it (A.R.); amusumeci@oasi.en.it (A.M.); afallea@oasi.en.it (A.F.); cali@oasi.en.it (F.C.); melia@oasi.en.it (M.E.); 2 Department of Biological, Geological and Environmental Sciences, University of Catania, Via Androne 81, 95124 Catania, Italy; concetta.federico@unict.it

**Keywords:** early-onset myopathy, ion channelopathy, P2X receptors, whole exome sequencing, *P2RX6* gene, skeletal muscle, electromyography, genetic diseases

## Abstract

Ion channelopathies result from impaired ion channel protein function, due to mutations affecting ion transport across cell membranes. Over 40 diseases, including neuropathy, pain, migraine, epilepsy, and ataxia, are associated with ion channelopathies, impacting electrically excitable tissues and significantly affecting skeletal muscle. Gene mutations affecting transmembrane ionic flow are strongly linked to skeletal muscle disorders, particularly myopathies, disrupting muscle excitability and contraction. Electromyography (EMG) analysis performed on a patient who complained of weakness and fatigue revealed the presence of primary muscular damage, suggesting an early-stage myopathy. Whole exome sequencing (WES) did not detect potentially causative variants in known myopathy-associated genes but revealed a novel homozygous deletion of the *P2RX6* gene likely disrupting protein function. The *P2RX6* gene, predominantly expressed in skeletal muscle, is an ATP-gated ion channel receptor belonging to the purinergic receptors (P2RX) family. In addition, STRING pathways suggested a correlation with more proteins having a plausible role in myopathy. No previous studies have reported the implication of this gene in myopathy. Further studies are needed on patients with a defective ion channel pathway, and the use of in vitro functional assays in suppressing *P2RX6* gene expression will be required to validate its functional role.

## 1. Introduction

Ion channelopathies encompass a broad spectrum of disorders arising from the impaired function of ion channel proteins, attributed to mutations in proteins that influence the regulation of ion transport across cell membranes [1,2]. Ion channels and transporters facilitate the movement of ions, playing a vital role in various organ functions. These channels conduct ions such as potassium (K^+^), sodium (Na^+^), and chloride (Cl^−^), which are crucial for regulating membrane potential [3,4]. Additionally, they transport hydrogen ions (H^+^) to manage intracellular and extracellular pH levels, as well as divalent cations like calcium (Ca^2+^), magnesium (Mg^2+^), and zinc (Zn^2+^), serving as second messengers and cofactors for numerous proteins [5].

In this framework, more than 40 different diseases have been associated with the ion channelopathy group, and it is worth mentioning peripheral neuropathy, chronic pain, migraine, epilepsy, and ataxia [6]. These disorders impact every major class of ion channel and affect all electrically excitable tissues, including the brain, peripheral nerve, skeletal and smooth muscle, and the heart [7].

Ion channelopathies have a significant impact on skeletal muscle (SM), which represents a contractile tissue consisting mainly of multinucleated myofibers. In this context, myopathy pertains to muscle disorders where the predominant symptom is muscle illness caused by fiber dysfunction [8,9]. Other symptoms include muscle cramps, stiffness (myotonia), spasms, and muscle fatigue, primarily affecting proximal muscle groups with a symmetric impairment distribution [10,11]. Myopathies are broadly categorized as inherited and/or acquired [12]. In particular, the most frequent inherited muscular myopathies include dystrophies, congenital metabolic myopathies, and myotonia [13,14]. Myopathy can be influenced by various factors, including conditions related to nucleotide disorders that affect energy production and ionic flow, such as ATP and NAD+. These disruptions can result in muscle and blood deficiencies [15]. Additionally, the development of myopathy can be attributed to the disruption of the network maintained by molecular chaperones, which play a protective role against the toxic misfolding and aggregation of proteins [16,17,18]. Chaperone dysfunction potentially belongs to pathogenic variants in genes encoding chaperones and co-chaperones of structural muscle [19].

As outlined by various authors, mutations in genes involved in the modulation of transmembrane ionic flow can be significantly associated with skeletal muscle disorders, particularly myopathies [20,21]. In fact, ion channels play a crucial role in regulating muscle excitability, the process of excitation–contraction coupling, and muscle contraction. A disturbance in the transmission of electrical signals can lead to depolarization at the motor endplate, thus triggering the activation of extra-synaptic voltage-gated sodium (Na^+^) channels. This alteration consequently leads to the generation of action potentials, calcium release (mainly from intracellular stores), and subsequent muscle contraction [22,23]. In addition, as previously described by several authors, mutations in genes related to chloride channels are closely linked to myopathies that disrupt permeation mechanisms, ultimately causing membrane depolarization [24].

Several muscular and neurological disorders characterized by motor symptoms have been associated with variations in the expression and function of purinergic receptors. Therefore, numerous studies have proposed a potentially robust association between disruption in purinergic receptors and the development of myopathy [25,26]. In fact, neuromodulators like purinergic ligands, adenosine, and ATP serve as signaling molecules with specific roles in different muscular pain syndromes. Consequently, the death of neural cells results in the uncontrolled release of ATP, subsequently impacting calcium signaling through the modulation of purinergic receptors [27]. Hence, reduced purinergic receptor function can trigger mitochondrial dysfunction, disrupting the oxidative phosphorylation process and causing oxidative burst, degranulation, and phagocytosis [28]. Additionally, this dysregulation can alter the levels of key second messengers like Ca^2+^ ions and reactive oxygen species, ultimately contributing to the development of myopathy and/or neurodegenerative disorders [29,30,31,32].

Electromyography is a valuable diagnostic tool, facilitating the precise differentiation of myopathy from other neuromuscular conditions through the evaluation of electrical activity in the muscles. Despite the electrophysiological evidence of a peripheral neuromuscular pathology, such electrophysiological examination does not always return the certainty of damage being primarily muscular or secondary to peripheral neuropathy. In the event that performing a muscle biopsy on the patient is impossible, the best possible diagnostic definition of neuromuscular diseases can be obtained through the use of next generation sequencing (NGS) approaches. In particular, NGS can significantly confirm diagnosis from electromyography or muscle biopsy [33,34].

Electromyography (EMG) test results are not always clear and the electrophysiological distinction between primary or secondary myopathy and axonal motor neuropathy may depend on the early or advanced phase of the disease. In the early phase of axonal motor neuropathy, EMG results may reveal a modest reorganization of the motor unit with a slight reduction in spikes (indicative of the number of motor units and lost axons) and short-lived polyphasic potentials, thus making it difficult to distinguish from a myopathy [35]. In the early phase of myopathy, EMG can show action potentials of normal duration, even in the advanced phase of the disease. Moreover, in the advanced phase of many myopathies, an EMG result of “single discharge” can be detected, similar to an axonal motor neuropathy. Even the detection of fibrillation potentials and mechanical irritability, found in distal muscles, is not pathognomonic of damage to the peripheral nerve, but they are frequently observed in slowly evolving myopathies and in the course of mild partial damage [36].

The activation of P2X receptors exerts multiple modulatory effects on synaptic plasticity [37]. These receptors comprise seven known mammalian subunits (P2X1–7) that form cation-selective channels activated by extracellular ATP. A receptor channel consists of three subunits, with each subunit being a polypeptide containing two transmembrane regions (TM1 and TM2), intracellular N- and C-termini, and a bulky extracellular region gripping adjacent subunits with extensive contact interfaces [38,39]. P2X receptors are distinctive proteins functioning as ligand-gated ion channels, triggered by ATP as a signaling molecule. They are predominantly located in skeletal muscle and are subject to regulation by p53, activating specific signaling pathways. These receptors are involved in regulating ion flow and stand apart from other neurotransmitter-gated ion channels in terms of their structural and functional characteristics [40,41,42,43]. P2X receptors are found in various tissues throughout the body. In certain smooth muscle cells, these receptors are responsible for mediating the rapid excitatory junction potential, inducing depolarization and muscle contraction [23]. In the central nervous system, the activation of P2X receptors facilitates the entry of calcium into neurons, triggering slower neuromodulator responses, such as the regulation of glutamate neurotransmitter receptor trafficking [44,45,46].

Currently, there are seven known subtypes of P2X ion channel receptors and eight subtypes of P2Y G-protein-coupled receptors. These receptors have implications in a wide range of muscular and neurological behavioral disorders, including Parkinson’s disease, Alzheimer’s disease, epilepsy, depression, autism, and pain [32,47,48]. Regarding the *P2RX6* gene, it is primarily expressed in skeletal muscle tissue, and its expression is under the regulation of the P53 gene [49,50]. A prior study, utilizing P2X-subunit-specific antibodies for immunolocalization and Western blotting, revealed the presence of P2X6 in nerves and the small artery tunica intima of control myofibers [50].

The present study describes a young female patient who complained of numbness and weakness exclusively in her arms, who underwent a thorough diagnostic assessment. Whole exome sequencing (WES) enabled the exclusion of other known genetic conditions compatible with the clinical and electrophysiological phenotype (like other myopathies or polyneuropathies). Conversely, the presence of a destructive mutation of the *P2RX6* gene, whose implication in muscle development and functioning is known, strengthens the hypothesis of genotype–phenotype correlation.

## 2. Materials and Methods

### 2.1. Electromyography

Electroneurography (ENoG) and electromyography (EMG) were conducted by using the instrument Medelec Synergy (Natus Medical Incorporated, Middleton, WI, USA). The analysis was performed in relation to both sensory and motor action potentials (SNAP and MAP, respectively) and (F) wave (conducted on the wrist). The parameters detected were conduction velocity (CV) (m s^−1^), amplitude (AMP) (mV), and latency (LAT) (ms). The EMG was performed with Dantec concentric needle, size 37 mm × 0.46 mm, area 0.07 mm^2^ (Natus Medical Incorporated, Middleton, WI, USA). The proximal and distal muscles of the bilateral upper limb and lower limb were explored. This study included resting activity, voluntary activity, and quantitative and qualitative analysis of MUPs (amplitude, duration, waveform).

### 2.2. Multiplex Ligation-Dependent Probe Amplification (MLPA) Analyses

Multiplex ligation-dependent probe amplification (MLPA) was performed for ruling out the possibility of spinal muscular atrophy (SMA) and Charcot–Marie–Tooth diseases. MLPA was conducted for evaluating SMA disease by using the SALSA P021-A2, P060-A1 kits and P460-A1 kit (MRC Holland, 1057 DL Amsterdam, The Netherlands). Conversely, CMT analysis was performed by using the SALSA MLPA kit P033, MRC-Holland. Analysis of MLPA data was performed by using Software Coffalyser NET v.220513 (MRC Holland, 1057 DL Amsterdam, The Netherlands). MLPA was carried out by using the Applied Biosystems Prism 3130 DNA Analyzer (Thermo Fisher Scientific, Waltham, MA, USA).

### 2.3. Preparation of Libraries and NGS Analysis

Genomic DNA was isolated from peripheral blood leukocytes obtained from the clinical case, as well as from the father and the mother. DNA extraction was performed by employing a higher yield protocol [51]. Exome analysis was performed using the Ion AmpliSeq™ Exome RDY Kits following the manufacturer’s instructions (Thermo Fisher Scientific, Waltham, MA, USA). The quality of libraries was assessed by using DNA 1000 chips on the Tape Station 4200 (Agilent, Santa Clara, CA, USA) and Qubit dsDNA BR Assay Kits (Invitrogen, Waltham, MA, USA). For the analysis, pooled libraries were employed to emulsion PCR on the Ion Chef Instrument according to the manufacturer’s protocol (Thermo Fisher Scientific, Waltham, MA, USA). Finally, each loaded Ion 550™ chip was sequenced on the S5 System (Thermo Fisher Scientific, Waltham, MA, USA). In total, 98% of regions of interest have a minimum coverage of at least 20×. The pathogenic variants were confirmed by conventional Sanger sequencing (Applied Biosystems Prism 3130 DNA Analyzer, Thermo Fisher Scientific, Waltham, MA, USA) using primers designed by the online tool available from the National Center for Biotechnology Information (NCBI) (https://www.ncbi.nlm.nih.gov/tools/primer-blast/ accessed on 15 July 2019). Sanger sequencing was additionally performed in a cohort of 40 asymptomatic unrelated individuals from the same geographical origin (Sicily, Italy) to rule out the presence of this rare mutation in healthy subjects.

### 2.4. Data Analysis

The structure of both the normal and truncated P2RX6 protein was assessed by using the UniProt database (https://www.uniprot.org/) and UCSF ChimeraX molecular modelling system for the high-quality protein visualization (accessed on 24 November 2023). The model related to the transmembrane localization of P2RX6 protein was obtained from the InterPro—EMBL-EBI (Pfam) database (https://www.ebi.ac.uk/, accessed on 24 November 2023). We excluded all the common variants, non-exonic polymorphisms, keeping polymorphisms with a minor allele frequency (MAF) of <1% in the public databases: gnomAD Exomes v.3.1.2, 1000 Genomes Project, and Exome Sequencing Project (accessed on 24 November 2023). The pathogenic variant was searched on The Human Gene Mutation Database (HGMD Professional 2023). VarAFT filtering (https://varaft.eu/ accessed on 29 April 2023) on vcf files was used. The variation found was classified according to the “American College of Medical Genetics” (ACMG) guidelines [52] and performed with VarSome [53]. Additionally, in silico evaluation of inheritance patterns for Mendelian conditions was analyzed by using the DOMINO tool (www.ub.edu/softevol/domino, accessed on 24 November 2023), according to prior studies [54,55]. In addition, the STRING database (https://string-db.org/, accessed on 24 November 2023) was employed to construct a functional protein association network.

## 3. Results

### 3.1. Case Description

We report the case of a 14-year-old female, the only child of non-consanguineous parents, delivered at term by cesarean section, as a prophylactic measure due to the mother’s epileptic condition, treated with Valproic acid (started at the seventh month of pregnancy). The birth weight was 3.3 kg, and no peri- or neonatal risk factors or family history of neurological diseases were reported. The patient achieved appropriate milestones in neuromotor development, sitting at 6 months and starting to talk and walk at around one year; menarche occurred at 11 years old. The patient experienced approximately four episodes of numbness and muscle weakness with easy fatigue in a single upper limb. The onset occurred over a few minutes, with symptoms appearing in a distal–proximal sense of variable duration, up to a maximum of 20 min.

Notably, no sensitivity disorders and signs of muscle hypotrophy were reported. Similar symptomatology was previously experienced in the ipsilateral lower limb. Organic or mechanical causes were ruled out by MR carried out on the cervical cord. Specifically, any compressive syndrome on the cord or on the roots of the spinal nerves was not detected. MR of the brain was also normal. Therefore, EMG and ENoG tests were performed on the patient. EMoG parameters, including examination of entrapment sites and F waves in the tested nerves, were all within normal ranges (Table 1).

Conversely, EMG analysis showed marked neurogenic damage with aspects of denervation (fibrillation potential) and signs of collateral reinnervation with MUP of slightly increased amplitude (4 mV) on the distal muscles (I dorsal interosseus). On the proximal muscles, EMG showed milder neurogenic suffering, without denervation, but with some muscle fields with motor unit potential (MUP) spikes of reduced duration (1–2 ms), as during primary muscle fiber damage. Furthermore, a significant increase in insertion activity and electrode needle movement was evident on almost all muscles explored, as “membrane instability”.

Toothagenesis (TA) in maxillary lateral incisors was observed. The NGS analysis revealed no causative variants underlying TA.

### 3.2. Next Generation Sequencing Analysis

**Figure 1:**
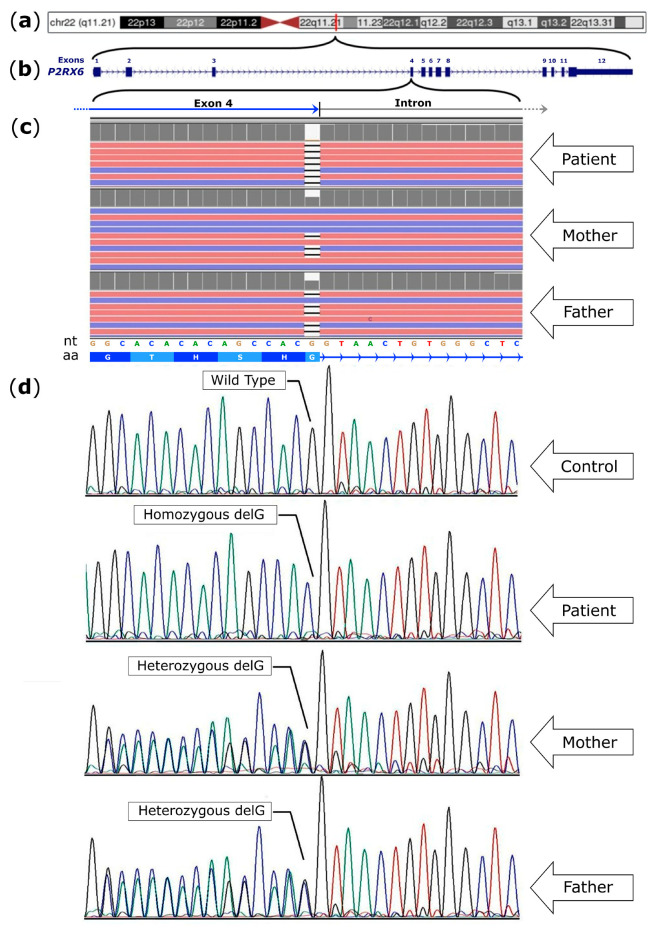
(**a**) Localization of *P2RX6* in chromosome 22 (band 22q11.21); (**b**) the *P2RX6* gene contains 12 exons and spans about 12 kb; (**c**) next generation sequencing (NGS) visualization with the IGV (Integrative Genomics Viewer) of the deletions of the G nucleotide in the *P2RX6* gene. Pink and blue reads are aligned to the forward and reverse strands, respectively. See also Table S1; nt: nucleotide sequence, aa: amino acid sequence; (**d**) Sanger sequencing of control, patient, mother, and father. The patient showed a homozygous deletion that resulted in a frameshift and premature termination codon (G155Vfs*2). The electropherograms show the sequences obtained with the reverse primer. Black, blue, green, and red profiles indicate G, C, A, and T nucleotide, respectively.

The NGS analysis uncovered a novel homozygous frameshift variant inherited from both healthy parents (Figure 1). This variant resulted from a Guanine (G) deletion at position 464 (c.464delG), leading to a disruption in the reading frame. Therefore, amino acids starting from position 155 (G155Vfs*2) are not translated, leading to the production of a shorter protein, i.e., approximately one-third of the wild-type protein. Furthermore, the NGS analysis did not reveal any additional variants within candidate genes associated with neuropathies.

**Figure 2:**
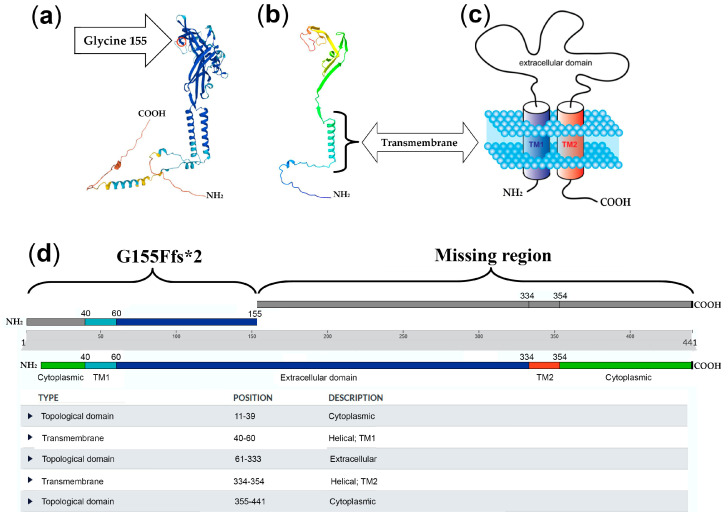
(**a**) P2RX6 protein model obtained by UniProt database (https://www.uniprot.org/, accessed on 24 November 2023); (**b**) in silico prediction model of truncated P2RX6 protein, achieved by the software UCSF ChimeraX v. 1.7 molecular modelling system; (**c**) model of the transmembrane localization of P2RX6 protein. The two transmembrane domains (TM1 and TM2) are evidenced. The model was obtained from the InterPro—EMBL-EBI (Pfam) database (https://www.ebi.ac.uk/, accessed on 24 November 2023); (**d**) deletion c.464delG (NM_005446.5) determined a shift in the reading frame in the *P2RX6* gene, generating a premature termination codon at the aa position 155 (G155Vfs*2). The truncated protein is considerably shorter than the wild type, 155 aa vs. 441 aa, determining the removal of the “extracellular topological domain” (61-333 aa), the second “transmembrane helix” (TM2) (334-354 aa), and the carboxyterminal “cytoplasmic domain” (355-441 aa).

The notable variation in the protein length of the mutated protein was from the standard 441 amino acids to a modified length of 155 amino acids (Figure 2). It is noteworthy that this novel mutation we detected is absent from the HGMD Professional Database 2023.3 (www.hgmd.cf.ac.uk accessed on 24 November 2023), LOVD 3.0, ClinVar, the 1000 Genomes Project, and the Exome Aggregation Consortium and GnomAD databases (accessed on 24 November 2023). Subsequently, Sanger sequencing of family members (patient, father, and mother) confirmed the presence of this mutation in the child affected by myopathic disease.

The analysis of 40 unrelated asymptomatic subjects (80 alleles) of the same geographical origin (Sicilian, Italy) by Sanger sequencing did not reveal the presence of this rare mutation.

## 4. Discussion

In the current work, we performed WES analysis on a girl whose EMG revealed muscular damage. The NGS analysis identified no additional variants within candidate genes associated with neuropathies or myopathy but revealed a novel homozygous deletion of the *P2RX6* gene disrupting protein function. In particular, the protein length of the mutated protein in both alleles was from the standard 441 amino acids to a modified length of 155 amino acids, removing the “extracellular topological domain” (61-333 aa), the second “helical transmembrane” (334-354 aa), and “cytoplasmic topological domain” (355-441 aa). Specifically, we posit that the removal of the extracellular domain specifically disrupts the interaction with extracellular ATP, thereby altering the ion flow mediated by the absent second transmembrane helix (TM2) of the P2X6 receptor. Within this context, Figure 2b depicts the prediction of the truncated protein, illustrating the absence of functional domains. Indeed, it is widely recognized that all purinergic receptors operate through the interaction with extracellular ATP, thus facilitating the activation of transmembrane ion channels [38,39].

Our findings highlight that the mode of inheritance appears to be autosomal recessive. Furthermore, the predictive analysis performed with DOMINO identified this variant as having an autosomal recessive inheritance pattern.

Notably, we are introducing, for the first time, a close association of the *P2RX6* gene with myopathy. Prior to our study, no documented human studies have reported this gene as causative for myopathy, as confirmed by the HGMD, ClinVar, and LOVD databases. The *P2RX6* gene is primarily expressed in the skeletal muscular systems, where it plays a key role in their development and function, interacting with various components such as muscle fiber, mitochondrial respiratory activity, and metabolic enzymes [42,56,57].

The examined patient presented numbness and muscle weakness in only one arm and one lower limb, suggesting a potential early-stage myopathy. Indeed, the EMG revealed the presence of neuromuscular involvement, supporting the hypothesis of a primarily muscular disease due to the absence of peripheral neuropathy (as confirmed by the negative response of the analyses of genes related to SMA and CMT) and the presence of motor unit potential (MUP) of reduced duration on the proximal muscles, which we believe to be an expression of an initial phase of the disease. The patient will undergo continuous monitoring in the coming years in order to assess the progressive severity of the muscular condition and potential onset of neuromuscular diseases later in life. It is worth noting that, despite the gene being predominantly expressed in skeletal muscle, we cannot exclude the possibility of late-onset neurological issues over time. The presence of more advanced damage on the distal muscles, where there are signs of reorganization of the MU, could be secondary to muscle degeneration [36].

Despite the evolution of molecular diagnosis and serology tests, muscle biopsy represents a crucial step in confirming myopathy diagnosis. Muscle biopsy plays a pivotal role in uncovering myopathy by providing crucial insights into the structural and functional aspects of muscle tissue [58]. It allows for the examination of muscle fibers, detection of abnormal cellular structures, and assessment of molecular markers, aiding in the accurate diagnosis and classification of myopathic conditions. In fact, muscle biopsy could document structural change or reduction in cytochromes or mitochondrial malfunction, adding the possible involvement of peripheral nervous system. This could be supported by a study which uses P2X-subunit-specific antibodies for immunolocalization and Western blotting, revealing P2X6 presence in nerves and small artery tunica intima of control myofibers [49].

Interestingly, both inherited and acquired myopathies often exhibit a gradual progression of symptoms, aligning with our preliminary observations [59]. EMG has proven to be useful in ruling out alternative phenotypes related to muscle disease and has highlighted its utility in refining accurate myopathy diagnoses.

Expanding on our findings, we propose that the dysfunctional production of the P2RX6 protein has an impact on ion channel transmembrane proteins. Specifically, these proteins have intricate associations with muscular and vascular diseases, as previously reported [60,61]. Remarkably, a causative connection was elucidated between abnormalities in transmembrane protein complexes responsible for transmembrane signal transmission and myopathy as well as other connective tissue diseases, manifesting effects on muscle structure [62,63,64].

Disruption of electrical signal transduction can lead to depolarization at the motor endplate, activating extrasynaptic voltage-gated sodium (Na^+^) channels, subsequently resulting in action potential, calcium release (primarily from intracellular stores), and muscle contraction [22,23]. The molecular mechanisms underlying ion channelopathies in myopathy involve the disruption of ion channels within muscle cells, resulting in impaired muscle function. Mutations in ion channel genes can interfere with downstream signaling cascades, impacting crucial processes like muscle growth, repair, and regeneration. These mutations may also alter membrane excitability, compromising muscle contraction and relaxation, ultimately causing muscle weakness or numbness [20]. Dysregulation of the above-mentioned processes can significantly influence the onset and progression of myopathy. Within this context, purinergic signaling, mediated by purine nucleotides like ATP and ADP, plays a pivotal role in various physiological processes, including muscle function. Alterations in P2X receptor expression, triggered by ATP, can potentially disrupt ATP-mediated signaling, resulting in impaired function within skeletal muscle fibers [65,66,67,68]. In this context, we propose that the novel mutation identified within the *P2RX6* gene has a significant impact on transmitter release at motor synapses. This alteration is presumed to affect the interplay between extracellular ATP and the trans-membrane localization of the TM2 functional domain due to incomplete protein translation. To further support our thesis, it is essential to underscore that the disruption of the potassium gate channel has a profound impact on the activity of various tissues, including skeletal muscle, neuronal brain, and the heart. Abnormalities in this channel have been shown to lead to muscular myopathy, as well as other diseases such as Huntington’s and dystonia [69,70,71,72]. Additionally, to strengthen our hypothesis, it is worth noting that potassium channels are intricately linked to both central and peripheral neuropathic pain [73,74].

The deletion we identified in the *P2RX6* gene may potentially impact this crucial ionic channel, contributing to the syndrome that we reported. Additionally, these defects can potentially impair cerebral endothelial and smooth muscle cells, contributing to further neurological impairments such as Alzheimer’s disease, developmental disorders, and epileptic encephalopathy [75,76]. Currently, the patient examined at the age of fifteen does not present neurological impairments.

Furthermore, a recent study revealed loss-of-function heterozygous variants resulting in truncating purinergic receptor proteins (P2RX6, P2RY4, P2RY11, P2RX4). These variations were found to be significantly enriched in patients with fibromuscular dysplasia (FMD) [77]. Nevertheless, fibromuscular dysplasia (FMD) and myopathy are distinct conditions, with FMD affecting arteries and myopathy involving muscle tissue dysfunction [78].

In addition, we conducted STRING analysis to identify proteins associated with our target gene *P2RX6*. This approach allowed for a comprehensive exploration of potential interactions, enhancing our understanding of the roles played by the P2RX6 protein in the mechanism involving molecular chaperones in neuromuscular diseases. Within this context, our analysis revealed correlations with several proteins, including BAG5, DNAJB4, and DNAJB5, which have plausible or confirmed roles in myopathy. The interaction among our target P2RX6 and the previously mentioned proteins was also revealed by high-throughput affinity-purification mass spectrometry, showing a robust protein–protein interaction [79]. In particular, BAG5 operates in mitigating mitochondrial dysfunction caused by oxidative damage [80] and plays a regulatory role in the nucleotide-binding domain responsible for ADP–ATP exchange [81].

Furthermore, DnaJ homolog, subfamily B, member 4 (DNAJB4) exhibited significant expression in myofibers. Dominant variants of DNAJB4 are associated with skeletal muscle and neuromuscular diseases [82,83]. Within this context, several references in the literature link the loss of function and dominant variants of DNAJB4 to myopathy [83,84,85]. In our research, we discuss the loss of function of P2RX6 to the dysregulation of signaling pathways, suggesting correlations with additional proteins also having potential roles in myopathy, including disturbances in mitochondrial oxidative processes. Indeed, we assume that the rare variant identified within the *P2RX6* gene may exert a potential effect on the pathway’s proteins. These effects could arise from the varied conformational molecular stoichiometry of the purinergic receptor or the alteration of other proteins’ activities as a consequence of the mutation. Furthermore, we hypothesize that the production of the truncated protein disrupts pivotal interactions within the previously elucidated metabolic and protein pathways. For instance, in vitro assays using patient-derived myoblasts could provide direct evidence of the mutation’s effect on muscle cell physiology. Therefore, more studies are needed on myopathic patients with a defective ion channel pathway, and the use of in vitro functional assays to knock out the expression of *P2RX6*, such as CRISP-R/Cas 9 and siRNA technologies, will be required to test the validity of the proposed pathogenic model. Moreover, functional studies on the P2RX6-related transcripts and proteins (BAG5, DNAJB4, and DNAJB5) are needed to elucidate and deepen our findings.

## 5. Conclusions

The primary objective of this research was to assess a potential correlation between the diagnosed myopathy in the patient and the homozygous (biallelic) deletion in the *P2RX6* gene, as revealed by WES analysis. Specifically, the truncated protein resulted in the absence of extracellular, transmembrane, and cytoplasmic domains, thereby altering ATP-gated ion channels. Notably, this gene is predominantly expressed in skeletal muscle. EMG allowed the detection of neuromuscular pathology and the exclusion of other muscle diseases, serving as a valuable diagnostic tool. Additionally, the examined patient exhibited numbness and weakness in only two limbs, suggesting an early-onset stage of myopathy. The patient’s clinical picture will be accurately monitored over time to evaluate the potential progression to a more severe form of myopathy in the future. Further investigations, including muscular biopsy, are essential to confirm the association of the *P2RX6* gene with myopathy, providing valuable insights into the structural and functional aspects of muscle tissue. This approach will further enhance our understanding of the complex interactions among genes encoding proteins within the ion channel signaling pathway, deepening the study of channelopathies.

## Figures and Tables

**Table 1:** Variation in the electromyography (EMG) parameters in the different nerves examined.

Nerves Examined	SNAP			MAP			F-Wave
	CV (ms^−1^)	AMP (mV)	LAT (ms)	CV (ms^−1^)	AMP (mV)	LAT (ms)	LAT (ms)
MEDIAN R (elbow-wrist)	62	126	2.7	60	10	3.4	26.3
ULNAR R (elbow-wrist)	61	86	2.8	55	13	2.9	28.7
ULNAR R (above-underelbow)	71	-	-	67	-	-	-
ESP R (knee-ankle)	-	-	-	51	5	2.6	42.4
SURAL R (calf. -ext. mall.)	60	25		-	-	-	

SNAP: sensory action potential; MAP: motor action potential; F-wave: fasciculation nerve response; CV: conduction velocity; AMP: amplitude; LAT: latency.

## Data Availability

The data presented in this study are available on request from the corresponding author.

## Supplementary Materials

The following supporting information can be downloaded at https://www.mdpi.com/article/10.3390/cimb46020073/s1: Table S1. Methodological details.
